# Structural basis of cyclic nucleotide selectivity in cGMP dependent protein kinase II

**DOI:** 10.1186/2050-6511-16-S1-A15

**Published:** 2015-09-02

**Authors:** James C Campbell, Kevin Y Li, Jeong Joo Kim, Gilbert Huang, Albert S Reger, Shinya Matsuda, Banumathi Sankaran, Todd M Link, Keizo Yuasa, John E Ladbury, Choel Kim

**Affiliations:** 1 Structural and Computational Biology and Molecular Biophysics Program, Baylor College of Medicine, Houston, Texas, USA; 2 Department of Biochemistry & Cell Biology, Rice University, Houston, USA; 3 Department of Pharmacology, Baylor College of Medicine, Houston, Texas, USA; 4 Verna and Marrs McLean Department of Biochemistry and Molecular Biology, Baylor College of Medicine, Houston, Texas, USA; 5 Department of Biological Science and Technology, The University of Tokushima Graduate School, Tokushima 770-8506, Japan; 6 Berkeley Center for Structural Biology, Lawrence Berkeley National Laboratory, Berkeley, California, USA; 7 Department of Biochemistry and Molecular Biology, The University of Texas MD Anderson Cancer Center, Houston, TX, USA

## Background

As a central mediator of the natriuretic peptide-cGMP signalling cascade, membrane bound type II cGMP dependent protein kinase (PKG II) is a key regulator of bone growth, renin secretion, and memory formation. It represents an important drug target for treating osteoporosis, cystic fibrosis, and memory loss [1-5]. In spite of its crucial physiological roles and its importance as a therapeutic target, little is known about its mechanisms of cyclic nucleotide selectivity and activation due to a lack of structural information. PKG II contains an N-terminal regulatory (R)-domain that binds a C-terminal catalytic (C)-domain in the absence of cGMP. Binding of cGMP to the cyclic nucleotide binding domains (CNB-A and B) within the R-domain releases the C-domain, leading to activation. We sought to understand the cyclic nucleotide selectivity and activation mechanisms of PKG II by studying each CNB domain.

## Methods and results

We screened and identified CNB domains of PKG II that are suitable for our structural studies using a high throughput Ligation Independent Cloning method. Our affinity measurements of the resulting CNB domains showed that CNB-B binds cGMP with a higher affinity, providing almost 500-fold selectivity, while CNB-A only offers 10-fold selectivity [6]. To understand the structural basis of each domain's cGMP selectivity, we solved crystal structures of CNB-A and -B in the presence of cyclic nucleotides. The structures revealed that only CNB-B shows an ordered C-helix that shields the cGMP pocket and specifically interacts with the guanine moiety through several hydrogen bond and VWD contacts. In contrast, CNB-A displays an open pocket without a C-terminal helix, resulting in fewer interactions with cGMP. Our mutation analysis demonstrated that the polar contacts at the C-helix of CNB-B are crucial for high cGMP selectivity and kinase activation.

## Conclusion

**Figure 1:**
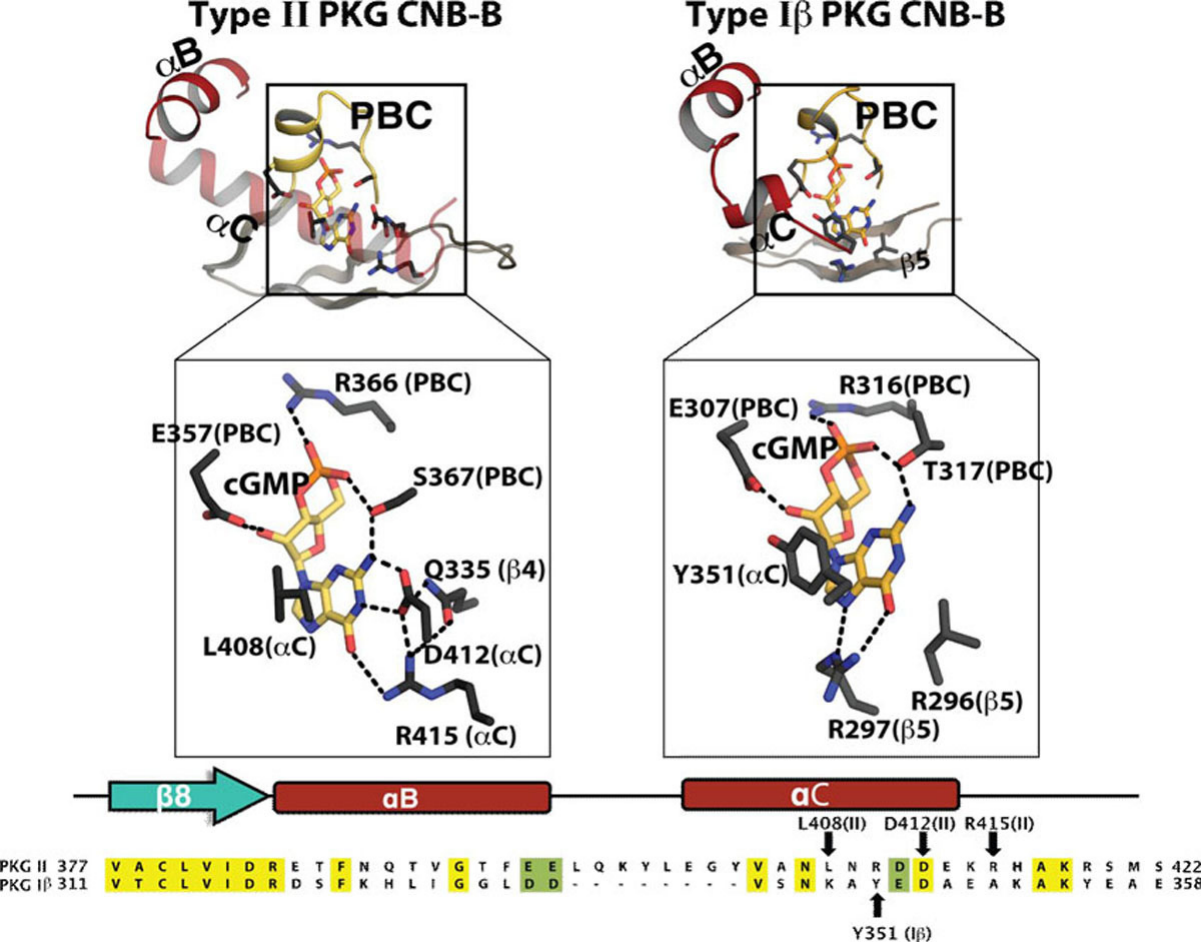
Isotype specific cGMP selectivity mechanisms of type I and II PKGs

Our structural comparison with cGMP selective PKG I CNB-B domain shows that it lacks cGMP specific hydrogen bonding contacts at the C-helix, which suggests a distinct cGMP selectivity mechanism for PKG II's CNB-B (Figure 1). Cyclic nucleotide compartmentalization is crucial for signalling specificity and exists in both cGMP and cAMP pathways [7-10]. Due to higher cAMP concentrations at the cell membrane compared to the cytosol, the higher cGMP selectivity seen in CNB-B of PKG II might be important in preventing activation of PKG II by cAMP, and this might minimize undesired cross-activation of both cyclic nucleotide signalling pathways.
